# Olfactomedin proteins: central players in development and disease

**DOI:** 10.3389/fcell.2014.00006

**Published:** 2014-02-26

**Authors:** Robert R. H. Anholt

**Affiliations:** Department of Biological Sciences and W. M. Keck Center for Behavioral Biology, North Carolina State UniversityRaleigh, NC, USA

**Keywords:** nervous system, myocilin, OLFM4, glaucoma, colorectal cancer

## Abstract

Olfactomedin proteins are characterized by a conserved domain of \texorpdfstring~\textasciitilde250 amino acids corresponding to the olfactomedin archetype first discovered in olfactory neuroepithelium. They arose early in evolution and occur throughout the animal kingdom. In mice and humans olfactomedin proteins comprise a diverse array of glycoproteins, many of which are critical for early development and functional organization of the nervous system as well as hematopoiesis. Olfactomedin domains appear to facilitate protein-protein interactions, intercellular interactions, and cell adhesion. Several members of the family have been implicated in various common diseases, notably myocilin in glaucoma and OLFM4 in cancer. This review highlights this important, hitherto understudied family of proteins.

## Introduction

**Figure 1:**
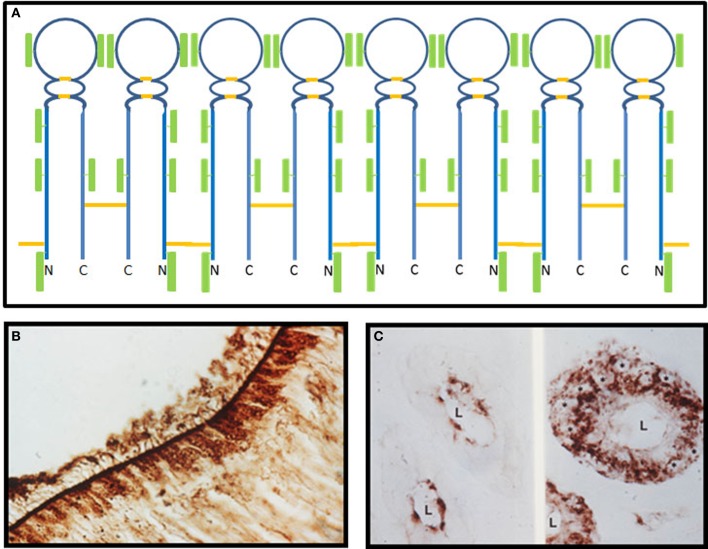
**Olfactomedin from the bullfrog. (A)** A model for the structure of olfactomedin based on biochemical and immunochemical studies. There are six N-glycosylated carbohydrate attachment sites, two internal disulfide bonds, and two cysteines, including the CXC motif near the N-terminus that can form intermolecular disulfides, which mediate the formation of oligomers through alternating single and double disulfide bonds. Disulfide bonds are shown in orange and the green rectangular boxes represent carbohydrate moieties (modified from Yokoe and Anholt, 1993). **(B)** Immunohistochemical localization of olfactomedin at the surface of the olfactory neuroepithelium of *Rana catesbeiana*. Granular staining of brown horseradish peroxidase reaction product can be seen in the sustentacular cells, and olfactomedin deposits are evident at the surface, where they associate with olfactory dendritic cilia (from Snyder et al., 1991). **(C)** Immunohistochemical localization of olfactomedin in acinar cells of Bowman's glands of the olfactory epithelium of *Rana catesbeiana*. The left panel shows brown horseradish peroxidase reaction product visualized with an antibody that recognizes the fully glycosylated protein, whereas the non-glycosylated precursor is detected by a different antibody in perinuclear regions of the acinar cells, shown in the right panel. The asterisks label the nuclei of acinar cells and L designates the lumen (from Bal and Anholt, 1993).

In the early 1990s, an abundant glycoprotein was discovered as a contaminant in chemosensory dendritic cilia preparations purified from olfactory epithelium of the bullfrog, *Rana catesbeiana* (Snyder et al., 1991). This protein is secreted into the nasal lumen, where it forms disulfide linked aggregates, which are localized to the lower mucus layer in intimate contact with chemosensory dendritic cilia (Bal and Anholt, 1993; Figure 1). Based on its distinct localization, it was named “olfactomedin” and hypothesized to serve as a differentiation signal for dendritic knobs of chemosensory neurons, which are replaced throughout adult life from stem cells in the base of the olfactory neuroepithelium (Snyder et al., 1991; Bal and Anholt, 1993; Yokoe and Anholt, 1993). This hypothesis has not yet been experimentally validated, but remains plausible, especially in the context of neurodevelopmental functions associated with related proteins, described in this review.

Olfactomedin was initially considered unique to olfactory neuroepithelium, but soon after its discovery alternatively spliced variants of a homolog—known initially as the AMZ protein, neuronal olfactomedin, and later as noelin—were found widely distributed throughout the rat brain and adrenal medulla (Danielson et al., 1994). The proteins from frog and rat show extensive homology, with conserved cysteines, including a CXC motif near the N-terminus, and six evenly distributed N-glycosylation sites at similar positions (Karavanich and Anholt, 1998). Biochemical studies with lectins and monoclonal antibodies showed evidence for intramolecular and intermolecular disulfide bonds, which enable the formation of homopolymers (Snyder et al., 1991; Figure 1). Subsequently, a vast number of proteins that share an ~250 amino acid domain homologous to olfactomedin and thought to mediate protein-protein interactions were discovered in animals ranging from nematodes to people (Zeng et al., 2005).

Olfactomedins appear to be critical mediators for development, including early development of the nervous system and hematopoiesis, and disruption of their expression results in dramatic developmental perturbations and lethality. Recapitulation of embryonic expression of some olfactomedin-domain proteins in adult life has been implicated in a variety of prevalent diseases, including glaucoma and cancer—most notably gastrointestinal cancers.

## The olfactomedin protein family

**Figure 2:**
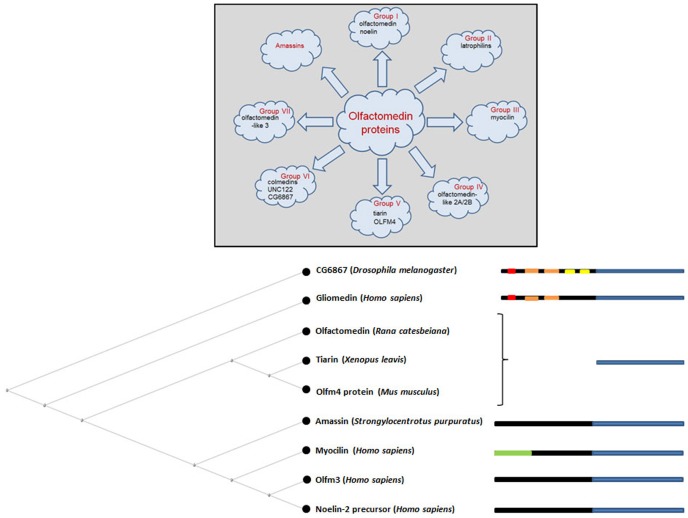
**Overview of the olfactomedin protein family**. The upper diagram highlights examples of olfactomedin proteins in each category focusing on proteins that are described in this review. The lower diagram shows a neighbor joining tree for some representative members of the family with diagrammatic representations of protein domains, indicating the olfactomedin homology domain in blue, collagen-like domains in orange, IgG-like domains in yellow, transmembrane domains in red, and the leucine zipper region in myocilin in green. Signal peptides and coiled-coil motifs in some members of the family, such as amassin, are not indicated. For complete phylogenetic analyses of the large superfamily of olfactomedin proteins, see Zeng et al. (2005) and Hillier et al. (2007).

Comprehensive phylogenetic analyses have categorized olfactomedin-domain proteins into seven subfamilies, designated with Roman numerals I through VII (Zeng et al., 2005; Figure 2). This classification used amassin, an olfactomedin-domain protein found in sea urchin (Hillier and Vacquier, 2003), as an outgroup. The identification of four additional sea urchin amassins, suggest that these olfactomedin-domain proteins should be considered a separate family, bringing the total number of subfamilies to eight (Hillier et al., 2007; Figure 2). Olfactomedin domains across all families show extensive sequence similarities, with signal peptides and invariant DExGLW and CG sequences in addition to cysteines and N-glycosylation sites shared among many members of the family at corresponding positions (Zeng et al., 2005). With the exception of subfamilies II and VI, olfactomedin domains are located at C-terminal regions. The original protein isolated from olfactory epithelium is a member of subfamily V and consists only of the canonical olfactomedin sequence. The most extensively studied member of the olfactomedin protein family is myocilin, which belongs to subfamily III, and is associated with glaucoma.

## Olfactomedins as mediators of development

### Role of olfactomedins in development and functional organization of the nervous system

Following the discovery of an olfactomedin homolog in the rat brain (Danielson et al., 1994), a screen of a cDNA library derived from quail neural crest and neural tube led to the identification of a similar protein (Barembaum et al., 2000). This protein showed a distinct expression gradient with highest expression in the neural fold region and lower expression at the ventral midline that correlated with closure of the neural tube during chick embryogenesis. It was named “noelin” based on the acronym “neuronal olfactomedin-related endoplasmic reticulum localized.” Retrovirus mediated overexpression of this protein promoted neural crest cell emigration and expanded the time span during which neural crest cells could emerge from the neural tube (Barembaum et al., 2000). Thus, studies on noelin provided the first direct evidence for a function of an olfactomedin-domain protein in neurogenesis.

A further study in *Xenopus* showed that the effects of an alternative splice form of noelin, noelin-1, extend beyond the formation of the neural crest and also impacts later stages of neural development (Moreno and Bronner-Fraser, 2001). Noelin is produced as four alternatively spliced isoforms which are differentially expressed at different developmental stages. One of those isoforms, noelin-4, inhibits differentiation of neural precursors, limiting the appearance of differentiated neurons even as the pool of neural precursors expands. The effects of noelin-4 could be counteracted by co-expression of noelin-1 and interactions between these isoforms may be critical for regulating the timing of neural differentiation during development (Moreno and Bronner-Fraser, 2005).

An additional member of the olfactomedin protein family in *Xenopus*, tiarin, has also been implicated in early formation of the nervous system (Tsuda et al., 2002). Unlike noelin, tiarin is expressed in non-neural head ectoderm that flanks the neural plate at the neurula stage of development and promotes dorsalization of the neural tube. Overexpression of tiarin results in induction of dorsal markers at the expense of ventral markers in the anterior central nervous system. The molecular mechanisms of action of noelin and tiarin remain to be determined, but initial studies on tiarin suggest that its action is independent of bone morphogenetic protein (BMP) or Sonic hedgehog (Shh) signaling associated with dorsalization and ventralization, respectively (Tsuda et al., 2002).

Similar neurodevelopmental functions of olfactomedin proteins have been observed in zebrafish. Overexpression studies and inhibition of protein expression by injection of morpholino antisense oligonucleotides in embryos showed that the zebrafish homolog of noelin, designated zebrafish olfactomedin 1, has profound effects on eye development, including eye size, the projection field of retinal ganglion cells to the optic tectum, and extension and branching of retinal ganglion cell axons (Nakaya et al., 2008). Co-immunoprecipitation experiments demonstrated a physical interaction between zebrafish olfactomedin 1 and WIF-1, a secreted inhibitor of the Wnt signaling pathway, suggesting that the effects of zebrafish olfactomedin 1 may be mediated through modulation of Wnt signaling (Nakaya et al., 2008). Further studies showed that zebrafish olfactomedin 1 promotes axon growth by activating the NogoA receptor complex (Nakaya et al., 2012).

**Figure 3:**
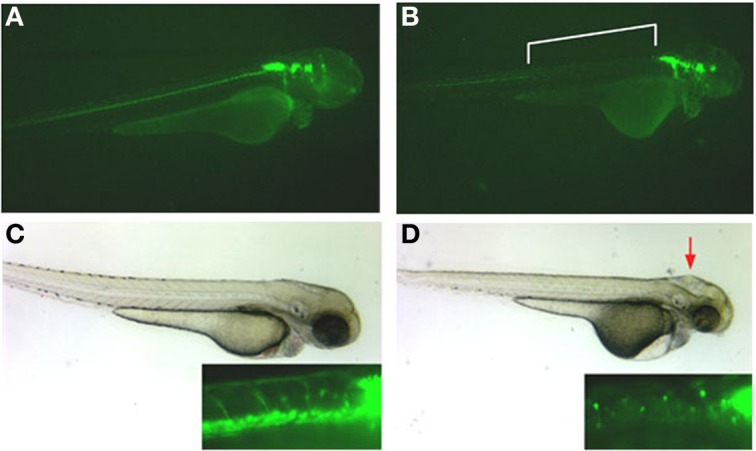
**Effect of olfactomedin 2 gene silencing on development of the nervous system in zebrafish**. Panels **(A,C)** show a 54 hpf normal embryo injected with a control morpholino-oligonucleotide that expresses GFP under the control of the Islet-1 promoter labeling the spinal cord and cranial nuclei. The inset in panel **(C)** shows axonal projections from spinal cord motor neurons and neatly aligned cell bodies of Rohon-Beard neurons. Panels **(B)** and **(D)** show embryos at the same stage injected with 5 ng of a morpholino-oligonucleotide that silences expression of the zebrafish olfactomedin 2 gene. Note the complete absence of anterior spinal cord motor neurons [bracketed area in panel **(B)** and inset in panel **(D)**] and jumbled locations of cell bodies of the Rohon-Beard neurons [inset in panel **(D)**]. Deformations of the head are also evident (red arrow). See also Lee et al. (2008).

Similarly, inhibition of protein expression of a second olfactomedin protein in zebrafish, zebrafish olfactomedin-2, by morpholino antisense oligonucleotides resulted in dramatic effects on development of the anterior central nervous system with craniofacial malformations attributable to perturbed differentiation of cranial neural crest cells (Lee et al., 2008; Figure 3), not unlike the effects of noelin and tiarin observed in *Xenopus* (Moreno and Bronner-Fraser, 2001; Tsuda et al., 2002). The olfactory pits and eyes were poorly developed, with the latter tending toward cyclopia, and the size of the optic tectum was greatly reduced. Rohon-Beard neurons in the anterior spinal cord appeared disorganized and axon guidance of branchiomotor neurons was disrupted (Lee et al., 2008; Figure 3).

**Figure 4:**
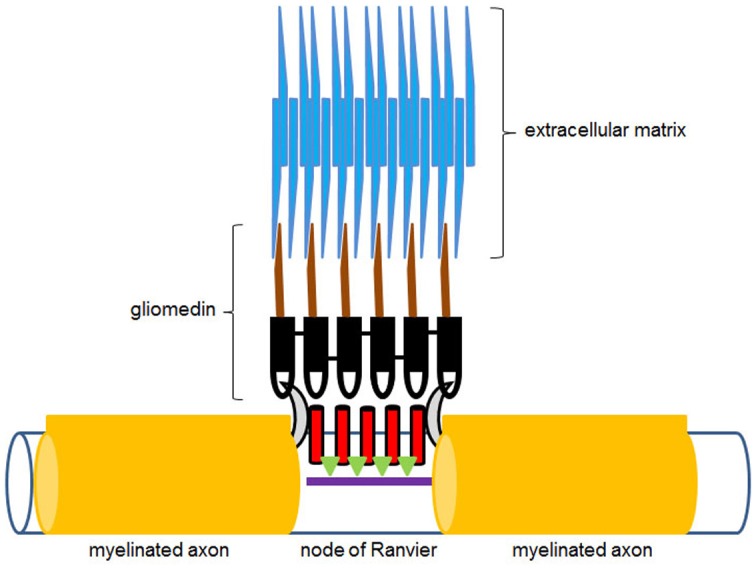
**Diagrammatic illustration of the hypothetical action of gliomedin at the nodes of Ranvier**. The olfactomedin domain of gliomedin, shown in black, interacts with neurofascin (gray half-moon shapes), while the collagen domain containing region of the molecule, shown in brown, is integrated in the extracellular matrix (blue). Voltage-dependent sodium channels at the nodes of Ranvier are shown in red. Green triangles represent ankyrin G, which links the channel proteins to β IV spectrin (purple), which is part of the axonal cytoskeleton underneath the nodes of Ranvier. See also Eshed et al. (2005).

In addition to mediating early stage neurogenesis, olfactomedin-domain proteins appear essential for enabling neural signal propagation. Elegant studies on rat dorsal root ganglion cell cultures showed that gliomedin, an olfactomedin-domain protein produced by Schwann cells, is essential for the formation of nodes of Ranvier and the clustering of voltage-dependent sodium channels during myelination (Eshed et al., 2005). Gliomedin is a transmembrane protein, which belongs to subfamily VI of the olfactomedin protein family (Figure 2). This family encompasses several invertebrate olfactomedin proteins, including the Drosophila *CG6867* gene product and the *Caenorhabditis elegans* UNC-122 protein (Zeng et al., 2005). These proteins are transmembrane proteins, which contain an extracellular olfactomedin domain along with collagen domains and are also referred to as “colmedins” (Loria et al., 2004). Proteolytic cleavage of gliomedin results in the release of a secreted extracellular fragment containing the olfactomedin domain (Eshed et al., 2007). This fragment undergoes homo-oligomerization, becomes integrated in the extracellular matrix in association with heparin sulfate proteoglycans, and binds to axonal Nr-CAM and fibronectin type III-like domains of neurofascin (Eshed et al., 2007; Labasque et al., 2011). This interaction is essential for the molecular assembly of the nodes of Ranvier (Figure 4). RNAi-mediated suppression of gliomedin expression prevents clustering of sodium channels, ankyrin G, and β IV spectrin, essential for the formation of nodes of Ranvier. The soluble olfactomedin domain alone appears to be sufficient to induce the formation of the nodes (Eshed et al., 2005).

Olfactomedin proteins are also implicated in synaptic stability. The UNC-122 protein in *C. elegans* is expressed postsynaptically at muscle synapses (Loria et al., 2004). Examination of *unc-122* mutants reveals abnormal sprouting of motor axons and locomotor defects, which become progressively worse as the animal ages. It has been proposed that UNC-122 is necessary for stabilizing synaptic transmission at the neuromuscular junction (Loria et al., 2004).

Similar indications of a trans-synaptic function for olfactomedin proteins come from the class II proteins, which includes the family of latrophilins (Matsushita et al., 1999; Figure 2). Latrophilin-1, also known as “calcium-independent receptor of α-latrotoxin (CIRL)” binds α-latrotoxin, a component of black widow spider venom, which results in massive exocytosis of presynaptic vesicles (Lelianova et al., 1997; Krasnoperov et al., 1999). Latrophilins are G-protein coupled receptors with an extracellular olfactomedin domain near the N-terminus. The olfactomedin domain is not necessary for α-latrotoxin mediated neurotransmitter release (Krasnoperov et al., 1999), but binds with high affinity to the presynaptic transmembrane adhesion molecule, neurexin (Boucard et al., 2012). Although the precise significance of this trans-synaptic bridging is not clear, it is reminiscent of the effects of the *C. elegans* UNC-122 protein at the neuromuscular synapse (Loria et al., 2004) and the effect of gliomedin in stabilizing nodes of Ranvier (Eshed et al., 2005).

Thus, a variety of olfactomedin proteins are associated with early differentiation of the nervous system as well as maintenance of neuronal stability. It appears that the former function is mediated primarily through secreted olfactomedin proteins, whereas the latter involves membrane-associated proteins with extracellular olfactomedin domains. Studies to date support the notion that olfactomedin domains mediate protein-protein interactions that serve to guide, establish, or maintain intercellular connectivity. The currently available information, however, represents only the tip of a slowly emerging iceberg. It remains yet to be determined precisely how temporal and spatial expression of different olfactomedin proteins is controlled, who all the possible interacting partners are for different members of this family, and which cellular pathways are triggered by these interactions.

### Human olfactomedin proteins

**Figure 5:**
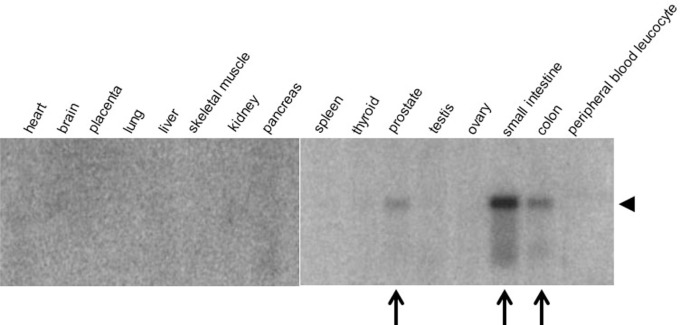
**Tissue-specific expression of OLFM4**. A radiolabeled oligonucleotide probe was hybridized to a Northern blot containing mRNA from human tissue samples. Note the specific expression of OLFM4 (arrowhead) in prostate, small intestine, and colon (arrows). Modified from Kulkarni et al. (2000).

Both mouse and human olfactomedin protein families consist of 13 gene products. A survey of 16 human tissues with probes corresponding to five olfactomedin-related proteins, OLFM1-OLFM4 (also designated as hOlfA-hOlfD) and myocilin, showed widespread and strikingly tissue-specific expression patterns (Kulkarni et al., 2000). OLFM1 and OLFM3 are neuronal olfactomedin proteins, with OLFM1 corresponding to the rat neuronal olfactomedin found throughout the brain and OLFM3, also known as optimedin (Torrado et al., 2002), expressed in cerebellum (Kulkarni et al., 2000) and in the eye (Torrado et al., 2002). In addition to its expression in brain, OLFM1 is also expressed in embryonic heart and has been implicated in heart development (Lencinas et al., 2013). OLFM2 is expressed in pancreas and prostate, and OLFM4 in colon, small intestine, and prostate (Kulkarni et al., 2000; Figure 5).

In addition to their role in neural development, olfactomedin proteins appear associated with immunity. Amassin in sea urchin and an olfactomedin homolog in *C. elegans* are expressed by coelomocytes, which fulfill an immune protective function in the body cavity (Hillier and Vacquier, 2003; Loria et al., 2004). Among human olfactomedins, such function seems to have been adopted by OLFM4, which in addition to its expression in the intestine and prostate is also expressed in bone marrow (Zhang et al., 2002). Here, pluripotent hematopoietic stem cells differentiate either via the lymphoid lineage into B- and T-cell derived lymphocytes or via the myeloid lineage into erythrocytes, granulocytes, macrophages, and platelets. OLFM4 (also designated GW112 or hGC-1, human Granulocyte Colony stimulating factor clone 1) is expressed during stem cell differentiation only in the myeloid lineage, where it becomes restricted to expression in granulocytes (Zhang et al., 2002; Rosenbauer et al., 2004). OLFM4 expression in myeloid precursor cells is controlled by phosphatidyl inositol 3 kinase and the ERK1/2 mitogen activated kinase pathway that activate the NF-κ B transcription factor, which binds to a promoter site in the 5′-upstream region of the OLFM4 gene (Chin et al., 2008). The precise function of OLFM4 in myeloid precursor cell differentiation remains as yet unknown.

## Olfactomedins and disease

### Myocilin and glaucoma

The most extensively studied olfactomedin protein to date is myocilin, which was first discovered in human trabecular meshwork cells (Stone et al., 1997). The trabecular meshwork is a connective tissue that regulates outflow at the iridocorneal angle of the eye and, hence, controls intraocular pressure. Ocular hypertension is a major risk factor for glaucoma, which results in progressive degeneration of the optic nerve. Diabetes mellitus is also known to confer risk for glaucoma and is accompanied by high circulating levels of the stress hormone cortisol. A major breakthrough in attempts to understand the etiology of glaucoma was the discovery that administration of cortisol to primary cell cultures of human trabecular meshwork cells resulted in pronounced overexpression of a protein, initially designated “trabecular meshwork inducible glucocorticoid response (TIGR) protein,” but more commonly known as “myocilin” (Stone et al., 1997; Clark et al., 2001).

Myocilin is a member of the olfactomedin protein family. It is a secreted glycoprotein that contains a C-terminal olfactomedin domain and an N-terminal leucine zipper motif (Nguyen et al., 1998). Mutations in its olfactomedin domain have been associated with more than 10% of cases of juvenile onset glaucoma and ~4% of cases of adult onset primary open angle glaucoma (Adam et al., 1997; Stone et al., 1997; Wiggs et al., 1998). Such association studies have been conducted and replicated in at least a dozen populations across the globe, making the association between myocilin polymorphisms and glaucoma risk one of the best consolidated relationships in human disease genetics (Anholt and Carbone, 2013). The importance of these discoveries cannot be overstated, as they provided the first insight into the molecular basis of a disease that on average affects 1 out of 100 individuals over the age of 40, with an even higher incidence among individuals of African and Hispanic descent (Quigley and Broman, 2006).

The myocilin gene consists of 3 exons and a 5-kb promoter region that contains 13 predicted hormone response elements, including glucocorticoid regulatory elements, and several additional regulatory motifs that point at complex regulation of its expression (Nguyen et al., 1998). Following translation, myocilin is cleaved in the endoplasmic reticulum by the calcium-dependent endoprotease calpain II at residues that link its leucine zipper motif and the C-terminal olfactomedin domain (Sánchez-Sánchez et al., 2007).

The function of myocilin is not known. There is evidence that myocilin can interact with optimedin (OLFM3), which is co-expressed with myocilin in trabecular meshwork and retina, and which is, based on its sequence, more closely related to noelin than myocilin (Torrado et al., 2002). Co-immunoprecipitation experiments demonstrated a direct interaction between these two secreted olfactomedin proteins (Torrado et al., 2002). Expression of optimedin, however, appears to be independent of glucocorticoids. Rather, the Pax6 transcription factor has been implicated in the regulation of optimedin expression (Grinchuk et al., 2005). In line with the cell adhesion properties of closely related olfactomedin proteins, optimedin promotes aggregation of NGF-stimulated PC12 cells and induces expression of N-cadherin (Lee and Tomarev, 2007). It is, therefore, conceivable that myocilin, either by itself or as a complex with optimedin, stabilizes intercellular interactions.

Based on the context with other olfactomedin proteins that mediate development of the nervous system, one can speculate that myocilin plays a role in development of eye structures and that recapitulation of excessive expression of myocilin under conditions of stress during adulthood has pathological consequences. A developmental function for myocilin is supported by evidence that myocilin may modulate Wnt signaling (Kwon et al., 2009) and that myocilin itself or its proteolytic fragments promote cell migration of NIH3T3 and FHL124 cells through activation of integrin focal adhesion kinase signaling (Kwon and Tomarev, 2011). Similarly, myocilin multimers, but not monomers, promoted substrate adhesion of trabecular meshwork cells in primary cell cultures (Goldwich et al., 2009).

**Figure 6:**
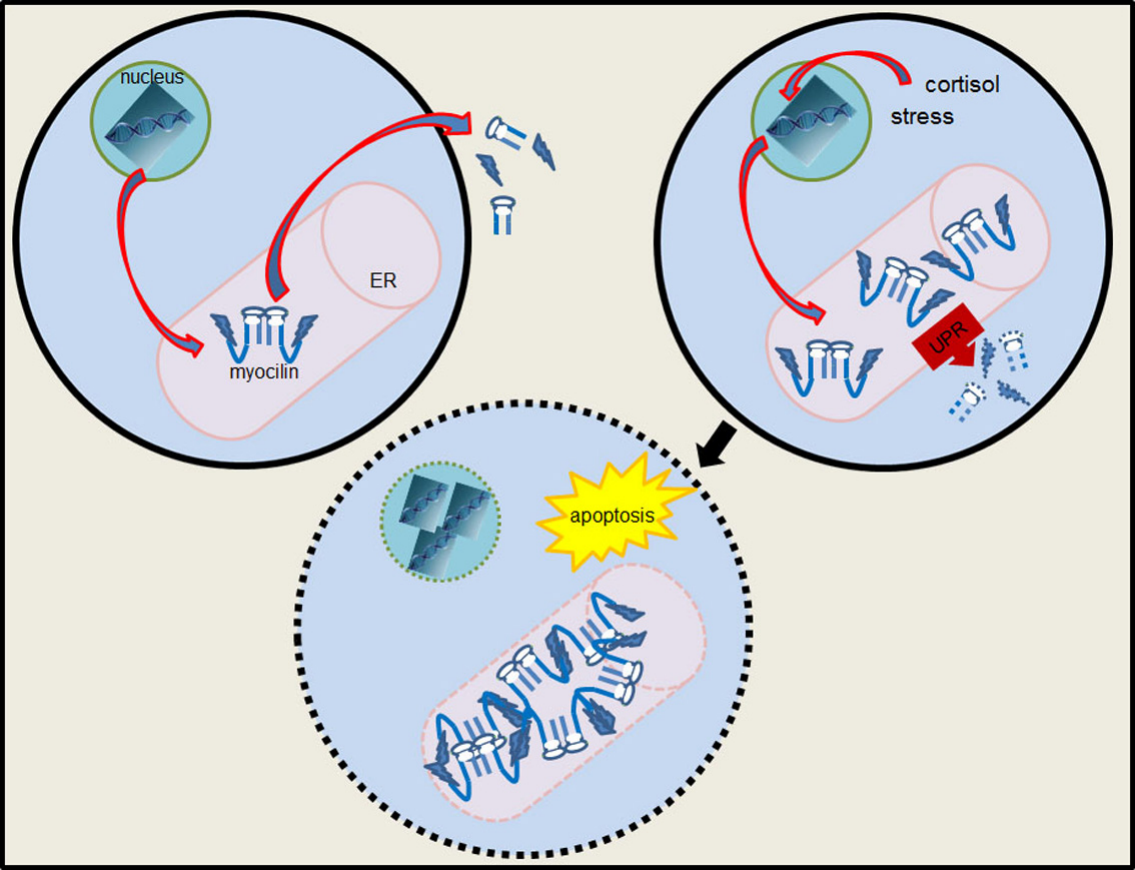
**Diagram of myocilin-induced endoplasmic reticulum stress and apoptosis**. Overexpression of myocilin results in the formation of aggregates in the endoplasmic reticulum (ER) and activation of the unfolded protein response (UPR). If accumulation of myocilin aggregates exceeds the capacity of the unfolded protein response the apoptotic pathway is activated. Jagged shapes represent the N-terminal domain of myocilin with leucine zipper motifs.

It is likely that the function of myocilin is unrelated to its relationship with glaucoma etiology. Truncated myocilin that lacks the olfactomedin domain and misfolded mutant myocilins with mutations in the olfactomedin domain are retained in the endoplasmic reticulum (Caballero et al., 2000; Gobeil et al., 2006). The latter also interfere with the secretion of optimedin (Torrado et al., 2002). It has been proposed that excessive accumulation of protein aggregates in the endoplasmic reticulum of trabecular meshwork cells leads to endoplasmic reticulum stress (Carbone et al., 2009; Anholt and Carbone, 2013). This would activate the unfolded protein response, a pathway that removes misfolded or aggregated proteins and processes them for proteolytic degradation via the proteasome (Hetz, 2012). Failure to accomplish this task would trigger apoptosis (Figure 6).

There is substantial evidence to support this hypothesis. Overexpression of human myocilin in the eye of *Drosophila melanogaster* results in an ocular hypertension phenotype in which ommatidia become distended and extrude fluid through the lenses onto the surface of the eye (Borrás et al., 2003). In the fly model overexpressed myocilin formed aggregates, and activation of the unfolded protein response could be visualized with a fluorescent xbp1-EGFP marker (Carbone et al., 2009). Subsequently, human homologs of Drosophila genes associated with the myocilin-induced unfolded protein response were found to harbor single nucleotide polymorphisms associated with primary open angle glaucoma in Salt Lake City (UT) and San Diego (CA) case-control study populations (Carbone et al., 2011). Replicate polymorphisms were identified in PDIA5 and BIRC6. PDIA5 encodes a protein disulfide isomerase that forms disulfide bonds during protein folding (Kozlov et al., 2010), whereas BIRC6 encodes an ubiquitin ligase that tags misfolded proteins for proteolytic degradation (Bartke et al., 2004).

There is additional independent support for involvement of the unfolded protein response pathway as a central mechanism for the pathogenesis of glaucoma. Accumulation of Y437H-myocilin, a mutant myocilin that causes severe glaucoma in people, in the endoplasmic reticulum of transgenic mice leads to activation of the apoptotic pathway and results in a glaucoma phenotype (Zillig et al., 2005; Zhou et al., 2008). Endoproteolytic cleavage of myocilin by calpain II prevents its aggregation and it has been suggested that misfolded mutant forms of myocilin render the calpain II cleavage site inaccessible, thereby enabling protein accumulation (Aroca-Aguilar et al., 2005).

In addition to its expression in the eye, myocilin is also found in skeletal muscle and heart (Kulkarni et al., 2000). Interestingly, no deficits in cardiac or muscle function have been reported for individuals carrying mutant myocilins that predispose to glaucoma.

### Olfactomedins and cancer

The role of olfactomedin proteins in cell adhesion and differentiation makes it intuitively plausible that they might function in cancer. Although no causal link has been demonstrated between altered expression of olfactomedin proteins and cancer, correlations between expression of OLFM4 (Figure 5) and cancer have been well documented. In addition to OLFM4, OLFM1 has been implicated in lung adenocarcinoma (Wu et al., 2010) and OLFM3 in endothelioma (Miljkovic-Licina et al., 2012), where it has been proposed to bind to BMP4 and enhance SMAD1/5/8 signaling required for BMP4-induced angiogenesis (Miljkovic-Licina et al., 2012). However, at present evidence for roles of OLFM1 and OLFM3 in cancer is scarce and needs to be further consolidated.

Most studies to date have focused on OLFM4, which has been associated with some of the most prevalent malignancies, including gastric cancer (Oue et al., 2009; Luo et al., 2011; Yu et al., 2011a; Liu et al., 2012), pancreatic cancer (Kobayashi et al., 2007; Chen et al., 2011), colon, lung and breast cancer, colorectal adenomas and liver metastases of colorectal origin (Seko et al., 2010; Besson et al., 2011; Sentani et al., 2013), endometriosis (Dassen et al., 2010), and cervical cancer (Yu et al., 2011b). Increased serum levels of OLFM4 have been proposed as diagnostic markers of early stages of gastric cancer and colorectal cancer (Oue et al., 2009; Grover et al., 2010; Seko et al., 2010; Yu et al., 2011a). In a study on 176 colorectal cancer patients, those in which positive cytoplasmic immunohistochemical staining for OLFM4 was observed had better survival rates than those who showed negative staining, suggesting that OLFM4 could serve as a potential prognostic marker for long term survival of colorectal cancer patients (Seko et al., 2010). OLFM4 expression is also up-regulated in gastric biopsies of patients with *Helicobacter pylori* infection and inflammatory bowel disease (Liu et al., 2010a). It is not clear to what extent up-regulation of OLFM4 under these conditions directly contributes to disease manifestation.

Human OLFM4 shows 65% amino acid identity to the canonical olfactomedin from olfactory epithelium of the bullfrog and like the frog olfactory protein is a secreted glycoprotein with six N-glycosylation sites (Grover et al., 2010). Like frog olfactomedin, OLFM4 forms large polymers through intermolecular disulfide bonds via a cysteine at position 226. Its glycoside moieties can bind to lectins on cell surfaces with high affinity (*K*_*D*_ = 1.57 × 10^−8^ M) and co-immunoprecipitation studies on transfected 293 T/17 cells showed that OLFM4 interacts with cadherin via its olfactomedin domain (Liu et al., 2006). Overexpression of OLFM4 in HT-29 colon carcinoma cells resulted in decreased cell adhesion and migration. However, OLFM4 gene sequences from 28 colon cancer samples did not show any sequence variants in a 1 kb promoter region or any of the five exons of the OLFM4 gene, although regulatory polymorphisms in intronic regions or epigenetic regulation cannot be excluded (Besson et al., 2011).

The normal function of OLFM4 in the gastro-intestinal tract is not known. The production of OLFM4 in the intestinal lining may fulfill an immune protective function. OLFM4 is highly expressed in a subset of pluripotent stem cells in the intestinal crypts, *Lgr5*^+^ cells, which express the Wnt target gene “leucine-rich repeat containing G-protein coupled receptor 5 (Lgr5)” (van der Flier et al., 2009). Its expression is here dependent on Notch signaling concomitant with cell proliferation (VanDussen et al., 2012). Several studies point at an anti-apoptotic function for OLFM4 (Liu et al., 2010b, 2012; Yu et al., 2011a), and a direct physical association between OLFM4 and the apoptotic protein GRIM-19 (retinoid-interferon-induced-mortality-19) has been reported (Zhang et al., 2004; Moreira et al., 2011). In this scenario, prevention of apoptosis by upregulation of OLFM4 in early stages of malignancy would enable cancer cells to survive and proliferate. Indeed, overexpression of OLFM4 in murine prostate cancer cells promoted tumor growth (Zhang et al., 2004). The physiological significance of an anti-apoptotic function of OLFM4 under normal conditions, however, needs to be further consolidated, as the primary role of OLFM4 is likely extracellular. One can speculate that the tumorigenic activity of overexpressed OLFM4 is dissociated from its normal physiological function and could be related to the induction of endoplasmic reticulum stress in parallel to the proposed mechanism by which myocilin accumulation may contribute to the etiology of glaucoma (Anholt and Carbone, 2013). This speculation presents a testable hypothesis for future studies.

## Future challenges

Since the discovery of the first olfactomedin, a large number of olfactomedin-domain proteins have been identified. However, a unifying view for the functions of these proteins and the rationale for the evolutionary conservation of the olfactomedin domain have not yet been formulated. It has become increasingly apparent that olfactomedins play essential roles in development and cell differentiation and that their effects are mediated through intercellular interactions, sometimes in conjunction with extracellular matrix components. Their expression is tissue-specific and regulation of their spatial and temporal expression is controlled by diverse transcriptional regulators. Mutations in critical regions of these proteins, including the olfactomedin domain, and perturbation of their expression levels are associated with several prevalent diseases, including glaucoma and cancer. Several unresolved questions remain for future studies. First, we need to determine the full spectrum of binding partners for all members of the olfactomedin superfamily. Also, the three-dimensional structure of the olfactomedin domain remains to be elucidated. What are the evolutionary forces that result in its evolutionary conservation, and how does variation within the conserved olfactomedin domain relate to its different binding partners? Our understanding of the precise mechanisms that regulate tissue-specific and temporally controlled expression of each of the olfactomedin proteins is still rudimentary at best and needs to be further explored. Another central question pertains to the cellular sequellae that are triggered by interactions between olfactomedin proteins and their binding partners. Furthermore, the precise functions of many olfactomedin proteins remain still unknown (e.g., the *Drosophila melanogaster CG6867* gene product). Finally, it is important to obtain a comprehensive assessment to what extent olfactomedin proteins are implicated in developmental disorders and to what extent variants in specific olfactomedin protein encoding genes contribute risk for diseases in addition to glaucoma.

It is likely that further studies might uncover additional contributions of olfactomedin proteins to diseases. Thus, this once neglected family of proteins must now be taken into account in biochemical studies of development and disease.

### Conflict of interest statement

The author declares that the research was conducted in the absence of any commercial or financial relationships that could be construed as a potential conflict of interest.
